# Elevated expression of TIGIT on CD3^+^CD4^+^ T cells correlates with disease activity in systemic lupus erythematosus

**DOI:** 10.1186/s13223-017-0188-7

**Published:** 2017-02-28

**Authors:** Qing Luo, Jianqing Ye, Lulu Zeng, Xue Li, Le Fang, Beihua Ju, Zikun Huang, Junming Li

**Affiliations:** 1 Department of Clinical Laboratory, The First Affiliated Hospital of Nanchang University, Nanchang, 330006 Jiangxi China; 2 Department of Medical College, Nanchang University, Nanchang, 330006 Jiangxi China; 3 Department of Blood Transfusion, 521 Hospital of Ordnance Industry, Xi’an, 710065 Shanxi China

**Keywords:** Systemic lupus erythematosus, TIGIT, T cells

## Abstract

**Objectives:**

It is well-known that lymphocytes play an important role in systemic lupus erythematosus (SLE). T cell immunoreceptor with Ig and immunoreceptor tyrosine-based inhibitory domains (TIGIT) is one of immunosuppressive costimulatory molecules that mediates an inhibitory effect. However, its roles in SLE are poorly understood. This study was designed to investigate the correlation between the frequencies of TIGIT-expressing CD3^+^CD4^+^ T lymphocytes and SLE.

**Methods:**

Patients with SLE were recruited from the First Affiliated Hospital of Nanchang University. Medical history, clinical manifestations, physical examination and laboratory measurements were recorded. The expression of TIGIT on CD3^+^ T lymphocytes, B lymphocytes, monocytes, neutrophils, CD3^+^CD4^+^ T lymphocytes and CD3^+^CD8^+^ T lymphocytes were determined by flow cytometry. The frequencies of TIGIT-expressing CD3^+^CD4^+^ T lymphocytes in patients with SLE were further analyzed for correlations with markers of autoimmune response, inflammation, urine proteins and disease activity in SLE.

**Results:**

The frequency of TIGIT-expressing CD3^+^CD4^+^ T lymphocytes was significantly elevated in SLE patients compared with healthy controls (P < 0.0001). The frequency of TIGIT-expressing CD3^+^CD4^+^ T lymphocytes in patients with SLE was increased significantly in subjects with high anti-dsDNA titer (P = 0.026), high anti-Sm titer (P = 0.026), and high levels of urine microalbumin (P = 0.046). Furthermore, The frequency of TIGIT-expressing CD3^+^CD4^+^ T lymphocytes was found to be positively correlated with the Disease Activity Index (SLEDAI) score in SLE (r^2^ = 0.082; P = 0.044).

**Conclusion:**

In SLE, the frequency of TIGIT-expressing CD3^+^CD4^+^ T lymphocytes was elevated and associated with the disease activity.

## Background

Systemic lupus erythematosus (SLE) is a prototypic systemic autoimmune disease characterized by the production of autoantibodies, deposition of immune complexes in various organs and potentially causing life-threatening renal, cardiac or brain damage [1, 2]. The mechanisms underlying SLE are complex, including genetic and environmental factors and abnormalities of both the innate and the adaptive immune systems [3]. Pivotal in the pathogenesis of lupus is the production of high-affinity pathogenic autoantibodies such as anti-double stranded DNA (anti-dsDNA) and anti-Smith (anti-Sm) [4]. Evidences from both human studies and animal models have demonstrated that both autoantibodies production and SLE pathogenesis are dependent on CD4^+^ T cells [5, 6]. Recently, some researches indicated that T cells with abnormal costimulatory molecules could activate autoantibody-producing B cells, suggesting the pivotal role of costimulatory molecules in the pathogenesis of SLE. Revealing the abnormalities of costimulatory molecule expression on immune cells is therefore crucial for understanding the mechanisms of SLE [7].

Costimulatory molecules have been proven to regulate the functional outcome of T cell activation. T cell immunoreceptor with Ig and immunoreceptor tyrosine-based inhibitory domains (TIGIT), also known as WUCAM, VSIG9 or VSTM3, is a newly identified inhibitory type 1 transmembrane protein. The poliovirus receptor (PVR, also known as CD155) has been identified as the physical ligand of TIGIT. The interaction of PVR, which is expressed mainly on antigen-presenting cells (APC), and TIGIT could mediate inhibitory effects on TIGIT-expressing cells [8]. In mouse model, loss of TIGIT resulted in hyperproliferative T cell responses and increased susceptibility to autoimmune diseases [9]. As expected, TIGIT was reported to inhibit the activation of T cells and NK cells, manifested by downregulating cytokines secretion by T cells and the cytotoxicity of NK cells [8, 10–12]. Furthermore, the levels of TIGIT on NK cells are significantly lower in patients with SLE than healthy individuals, and associated with the increase of IFN-γ-producing NK cells in patients with SLE [13]. However, the expression and roles of TIGIT on other immune cells in the condition of SLE are unclear yet.

In the present study, we detected the expression of TIGIT on peripheral blood leucocytes and quantified the proportions of TIGIT-expressing peripheral T subset cells in patients with SLE. The correlation between the frequency of TIGIT-expressing CD3^+^CD4^+^ T lymphocytes and the activity of SLE was also evaluated.

## Methods

### Subjects

50 patients fulfilled the revised American College of Rheumatology criteria for SLE [14] were enrolled from the First Affiliated Hospital of Nanchang University from June 2015 to December 2015. Disease activity was assessed by the SLE disease activity index (SLEDAI) [15, 16]. In addition, this study included 27 healthy controls (HCs) who were unrelated to the patients and who did not have inflammatory or autoimmune diseases. The study was approved by the Ethics Committee of the First Affiliated Hospital of Nanchang University (052) and was carried out in compliance with the Helsinki Declaration. Informed consent was obtained from all participants before they entered the study.

### Flow cytometry analysis

Fresh peripheral blood specimens were collected from patients with SLE and HCs. The molecular phenotypes of peripheral blood leucocytes were analyzed immediately using flow cytometry. The following antibodies were used: ECD-conjugated anti-CD3, PC5-conjugated anti-CD8, FITC-conjugated anti-CD4, PC5-conjugated anti-CD15, ECD-conjugated anti-CD14, PC5-conjugated anti-CD19 (BD Biosciences, San Diego CA, USA) and PE-conjugated anti-TIGIT (MIH clones, e Bioscience, San Diego, CA, USA). Briefly, 100 μL of fresh peripheral blood was incubated simultaneously with 10 μL of ECD-conjugated anti-CD3, 10 μL of PC5-conjugated anti-CD8, 10 μL of FITC-conjugated anti-CD4 and PE-conjugated anti-TIGIT or with 10 μL of ECD-conjugated anti-CD3, 10 μL of PC5-conjugated anti-CD15 and PE-conjugated anti-TIGIT or with 10 μL of ECD-conjugated anti-CD14, 10 μL of PC5-conjugated anti-CD19 and PE-conjugated anti-TIGIT on ice in the dark for 30 min. Cells incubated with PE-conjugated mouse IgG were used as isotype controls. RBCs were lysed with an ammonium-chloride-potassium lysing buffer, and samples were washed and analyzed using a CYTOMICS FC 500 flow cytometer (BECKMAN COULTER) and associated software programs (CXP).

### Autoantibody measurement

Anti-dsDNA of IgG in serum were measured using commercially available ELISA kits (Kexin, Shanghai, China). Anti-extractable nuclear antigens (ENAs) antibodies including anti-SSA, anti-SSB, anti-Ro52, anti-Sm, anti-nRNP/Sm, anti-rRNP, and anti-nucleosome antibody were determined using immunoenzyme dot assay (Euroimmun, Germany) according to the manufacturer’s instructions. The results of anti-ENAs detection were showed in negative (−) and positive (+, ++, +++) manner by EuroBlot One.

### Serum IgG, C3, C4 and urine protein measurement

The concentrations of serum immunoglobulin G (IgG), Complement 3 (C3), Complement 4 (C4) and urine protein including urine a_1_-microglobulin (a_1_-M), urine microalbumin (MALB), urine IgG (IGU) and urine transferring (TRU) were determined using nephelometry methods according to the instructions described by the manufacturer (IMMUNE800, Beckman, American).

### ESR, urine routine, blood routine measurement

Erythrocyte sedimentation rate (ESR), urine routine and blood routine were determined according to the instructions described by the manufacturer.

### Statistical analysis

Statistical analysis and graphic presentation were carried out with GraphPad Prism version 5.0 (GraphPad Software, San Diego, CA). A t test was used if a normality test was passed; otherwise, the nonparametric Mann–Whitney test was used to analyze the data. Likewise, the Pearson method or the nonparametric Spearman method was used for correlation analysis. A P value of <0.05 was considered significant.

## Results

### Characteristics of study subjects

The characteristics of SLE patients and healthy subjects (HCs) enrolled in this study are shown in Table 1. There were no significant differences between patients and HCs regarding age or gender. Patients with SLE were classified into inactive group (SLEDAI: 0–9) and active group (SLEDAI ≥ 10) according to SLEDAI [15, 16]. Overall, 32% of SLE patients are active patients. The two groups differ significantly in SLEDAI score, incidence of serum levels of anti-dsDNA, C3 and the proportion of circulating lymphocytes (all P < 0.05) (Table 2). Among them, 16 patients were new-onset SLE. The new-onset SLE and re-visiting SLE groups differ significantly in incidence of serum levels of anti-dsDNA, C3 and ESR (all P < 0.05) (Table 3).

**Table 1:** Clinical characteristics and proportions of lymphocyte subsets in peripheral blood of patients with SLE and HCs

Categories	SLE (n = 50)	HCs (n = 27)
Females, n (%)	46 (92)	23 (85)
Age, mean (SD), years	35.5 ± 13.8	33.7 ± 10.8
SLEDAI score, mean (SD)	6.3 ± 4.9	–
ANA (+, ≥1:100), n (%)	49 (98.0)	–
ds-DNA (+, >100 IU/mL), n (%)	19 (38.0)	–
Anti-ENA (44 patients)		
Anti-Sm, n (%)	18 (40.9)	–
Anti-Ro52, n (%)	28 (63.6)	–
Anti-nRNP/Sm, n (%)	28 (63.6)	–
Anti-rRNP, n (%)	21 (47.7)	–
Anti-nucleosome, n (%)	16 (36.4)	–
Anti-SSA, n (%)	37 (84.1)	–
Anti-SSB, n (%)	11 (25.0)	–
Decreased C3/C4, n (%)	37 (74.0)/30 (60.0)	–
Increased IgG, n (%)	26 (52.0)	–
Elevated ESR, n (%)	29 (58.0)	–
Urine protein (20 patients)		
a_1_-M > 12.5 mg/L, n (%)	13 (65.0)	
MALB > 20 mg/L, n (%)	11 (55.0)	
IgU > 8 mg/L, n (%)	11 (55.0)	
TRU > 2 mg/L, n (%)	9 (45.0)	
Clinical features		
Fever, n (%)	8 (16.0)	–
Cutaneous manifestations, n (%)	18 (36.0)	–
Oral ulcer, n (%)	5 (10.0)	–
Alopecia, n (%)	10 (20.0)	–
Arthritis, n (%)	16 (32.0)	–
Raynaud’s phenomenon, n (%)	9 (18.0)	–
Effusion, n (%)	6 (12.0)	–
Renal involvement, n (%)	19 (38.0)	–
Hematologic disorder, n (%)	32 (64.0)	–
Lymphocytes, mean (SD), %^a^	17.2 (9.4)*	23.7 (6.7)
CD3^+^ lymphocytes, mean (SD), %^b^	69.9 (12.2)*	63.4 (7.8)
CD4^+^ T subset, mean (SD), %^c^	45.9 (12.9)*	54.4 (9.2)
CD4^+^ T subset, mean (SD), /mL	313.4 (34.2)*	423.4 (31.3)
CD8^+^ T subset, mean (SD), %^c^	38.2 (12.8)*	28.9 (6.5)
CD8^+^ T subset, mean (SD), /mL	419.4 (59.1)	432.4 (64.9)
Ratio of CD4^+^/CD8^+^T, mean (SD)	0.98 (0.58)*	1.29 (0.52)

*a*_*1*_-*M* urinea_1_-microglobulin, *ANA* anti-nuclear antibodies, *Anti*-*dsDNA* anti double-stranded DNA, *Anti*-*SSA* anti-SSA antigen, *Anti*-*SSB* anti-SSB antigen, *C3* complement 3, *C4* complement 4, *ESR* erythrocyte sedimentation rate, *HCs* healthy controls *IgG* immunoglobulin G, *IGU* urine IgG, *MALB* urine microalbumin, *RNP* ribonucleoprotein, *rRNP* ribosomal RNP, *SLE* systemic lupus erythematosus, *SLEDAI* SLE disease activity index, *Sm* Smith, *TRU* urine transferrin

* P < 0.05 compared to healthy control group

^a^Percentage of total white blood cells in peripheral blood

^b^Percentage of total lymphocytes in peripheral blood

^c^Percentage of CD3^+^ lymphocytes in peripheral blood

**Table 2:** Demographic and clinical data for Chinese patients with active or inactive SLE

Categories	Active SLE	Inactive SLE
N	16	34
Females, n (%)	16 (100)	30 (88.2)
Age, mean (SD), years	32.8 (9.4)	36.7 (15.1)
ANA (+, ≥1:100), n (%)	16 (100)	33 (97.1)
Anti-dsDNA, mean (SD), IU/mL	355.8 (328.4)*	148.4 (239.9)
IgG, mean (SD), g/L	19.6 (9.8)	16.8 (7.9)
C3, mean (SD), g/L	0.45 (0.23)*	0.66 (0.26)
C4, mean (SD), g/L	0.11 (0.062)	0.14 (0.060)
ESR, mean (SD), mm/h	63.1 (44.1)	34.2 (35.5)
SLEDAI score, mean (SD)	12.6 (2.4)*	3.6 (2.8)
Lymphocytes, mean (SD), %^a^	12.0 (1.7)*	19.5 (1.9)
CD3^+^ lymphocytes, mean (SD), %^b^	73.2 (2.7)	68.2 (2.5)
CD4^+^ T subset, mean (SD), %^c^	42.5 (4.4)	47.3 (2.7)
CD4^+^ T subset, mean (SD), /mL	243.5 (69.6)	343.4 (38.2)
CD8^+^ T subset, mean (SD), %^c^	59.1 (4.0)	53.1 (2.6)
CD8^+^ T subset, mean (SD), /mL	336.1 (80.0)	455.1 (77.1)
Ratio of CD4^+^/CD8^+^ T, mean (SD)	0.81 (0.17)	1.12 (0.15)

*ANA* anti-nuclear antibodies, *Anti*-*dsDNA* anti double-stranded DNA, *C3* complement 3, *C4* complement 4, *ESR* erythrocyte sedimentation rate, *IgG* immunoglobulin G, *SLE* systemic lupus erythematosus, *SLEDAI* SLE disease activity index

* P < 0.05 compared to inactive SLE group

^a^Percentage of total white blood cells in peripheral blood

^b^Percentage of total lymphocytes in peripheral blood

^c^Percentage of CD3^+^ lymphocytes in peripheral blood

**Table 3:** Demographic and clinical data for Chinese patients with new-onset and re-visiting SLE

Categories	New-onset SLE	Re-visiting SLE
N	16	34
Females, n (%)	15 (93.8)	31 (91.4)
Age, mean (SD), years	28.1 (11.1)	38.2 (14.6)
ANA (+, ≥1:100), n (%)	16 (100)	33 (97.1)
Anti-dsDNA, mean (SD), IU/mL	346.6 (329.3)*	165.3 (267.7)
IgG, mean (SD), g/L	19.1 (5.9)	17.0 (9.6)
C3, mean (SD), g/L	0.52 (0.33)	0.63 (0.23)
C4, mean (SD), g/L	0.09 (0.060)*	0.16 (0.050)
ESR, mean (SD), mm/h	62.1 (41.2)*	35.1 (38.0)
SLEDAI score, mean (SD)	8.4 (5.0)	5.5 (4.8)
Lymphocytes, mean (SD), %^a^	17.1 (1.0)	16.8 (7.9)
CD3^+^ lymphocytes, mean (SD), %^b^	68.3 (11.0)	70.3 (9.9)
CD4^+^ T subset, mean (SD), %^c^	40.8 (5.4)	47.8 (14.9)
CD4^+^ T subset, mean (SD), /mL	269.5 (8.7)	330.1 (239.4)
CD8^+^ T subset, mean (SD), %^c^	59.2 (5.4)	52.2 (14.9)
CD8^+^ T subset, mean (SD), /mL	435.8 (97.5)	455.1 (77.1)
Ratio of CD4^+^/CD8^+^T, mean (SD)	1.02 (0.85)	1.03 (0.64)

*ANA* anti-nuclear antibodies, *Anti*-*dsDNA* anti double-stranded DNA, *C3* complement 3, *C4* complement 4, *ESR* erythrocyte sedimentation rate, *IgG* immunoglobulin G, *SLE* systemic lupus erythematosus, *SLEDAI* SLE disease activity index

* P < 0.05 compared to re-visiting SLE group

^a^Percentage of total white blood cells in peripheral blood

^b^Percentage of total lymphocytes in peripheral blood

^c^Percentage of CD3^+^ lymphocytes in peripheral blood

### T lymphocyte subsets in patients with SLE and HCs

While SLE patients showed a significantly lower proportion of circulating lymphocytes than HCs (17.2 vs 23.7%; P = 0.0053) (Table 1), the proportion of CD3^+^ T lymphocytes in total circulating lymphocytes was higher in SLE patients than HCs (69.9 vs 63.4%; P = 0.016) (Table 1). Further analysis showed that, the proportion of CD3^+^CD4^+^ T lymphocytes in CD3^+^ lymphocytes was significantly decreased in patients with SLE compared to HCs (45.9 vs 54.4%; P = 0.0094) (Table 1), and the proportion of CD3^+^CD8^+^ T lymphocytes in CD3^+^ lymphocytes was significantly elevated in patients with SLE compared to HCs (38.2 vs 28.9%; P = 0.0093) (Table 1). Moreover, the absolute CD3^+^CD4^+^ T lymphocytes numbers was significantly decreased in patients with SLE compared to HCs (313.4 vs 446.2; P = 0.0042) (Table 1), but no difference was found in the absolute CD3^+^CD8^+^ T lymphocytes numbers (Table 1). As a result, the ratio of CD3^+^CD4^+^ T cells to CD3^+^CD8^+^ T cells was significantly lower in SLE patients (0.98 vs 1.29; P = 0.043) (Table 1).

### The frequency of TIGIT-expressing peripheral blood leucocytes in SLE patients and HCs

**Fig. 1:**
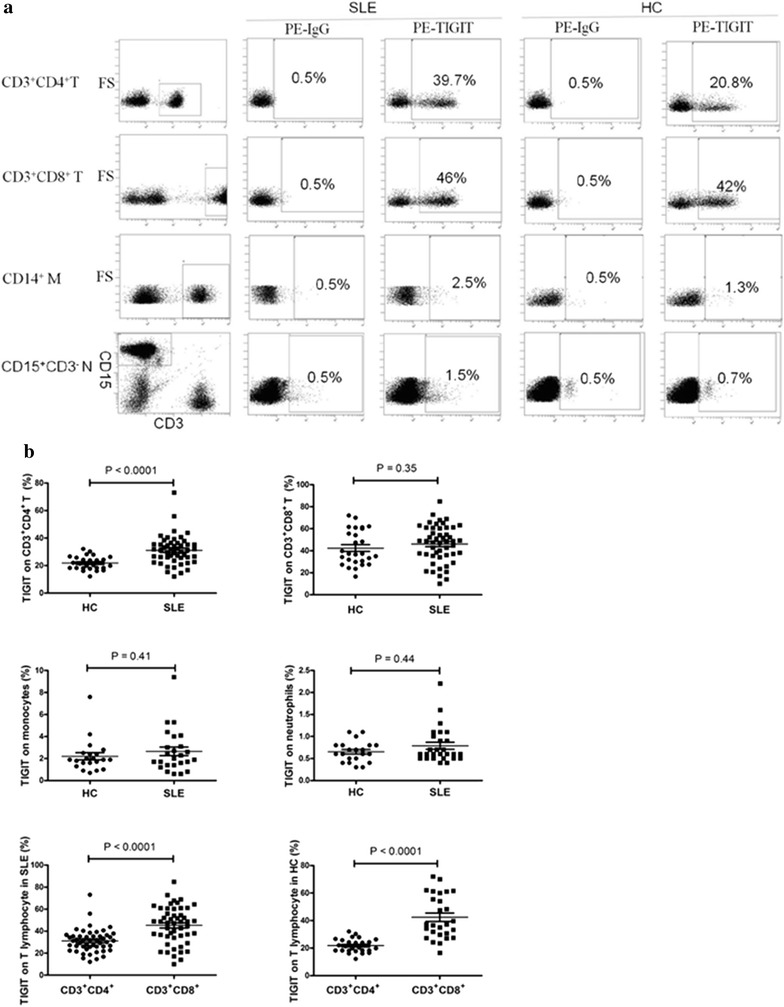
TIGIT expression on T lymphocytes subsets, monocytes and neutrophils. **a** Representative dot plots of population gating and TIGIT expressing cells from SLE patients and control subjects. Percentages of TIGIT expressing cells among CD3^+^CD4^+^, CD3^+^CD8^+^, CD14^+^ or CD15^+^CD3^−^ cells are shown. **b** Summary data of the positive cell frequency in gated CD3^+^CD4^+^ cells, CD3^+^CD8^+^ cells, monocytes or neutrophils

To determine the expression profile of TIGIT in SLE patients and HCs, we used flow cytometry to assess the expression of TIGIT on peripheral blood leucocytes including CD3^+^CD4^+^ T lymphocytes, CD3^+^CD8^+^ T lymphocytes, B lymphocytes, monocytes and neutrophils. Data showed that although the absolute number of TIGIT-expressing CD3^+^CD4^+^ T lymphocytes decreased, the frequency of TIGIT-expressing CD3^+^CD4^+^ T lymphocytes was significantly elevated in patients with SLE compared to HCs (P < 0.0001) (Fig. 1). No significant difference was observed in the frequency of TIGIT-expressing CD3^+^CD8^+^ T lymphocytes, monocytes and neutrophils between SLE individuals and HCs (Fig. 1). And, B lymphocytes had no apparent TIGIT expression. Further, results showed the frequency of TIGIT-expressing CD3^+^CD8^+^ T lymphocytes was significantly elevated compared to CD3^+^CD4^+^ T lymphocytes in both SLE patients (P < 0.0001) (Fig. 1) and HCs (P < 0.0001) (Fig. 1). The mean fluorescence intensity (MFI) of TIGIT in CD3^+^CD4^+^ T lymphocytes and CD3^+^CD8^+^ T lymphocytes from SLE patients and HCs was also determined, but no significant difference was found (data not shown).

### The frequency of TIGIT-expressing CD3^+^CD4^+^ T lymphocytes correlated with markers of T cell activation and other costimulatory molecules

**Fig. 2:**
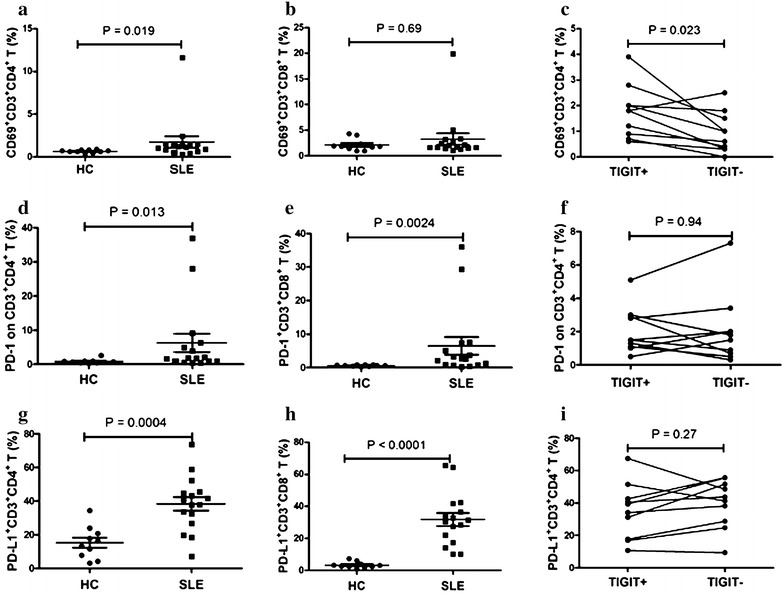
Correlation of frequency of TIGIT-expressing CD3^+^CD4^+^ T lymphocytes with markers of T cell activation and other costimulatory molecules. **a** The frequency of CD69-expressing CD3^+^CD4^+^ T lymphocytes was significantly increased in SLE patients compared to HCs (P = 0.019). **b** The frequency of CD69-expressing CD3^+^CD8^+^ T lymphocytes was similar between HCs and SLE (P = 0.69). **c** The frequency of CD69-expressing CD3^+^CD4^+^ TIGIT^+^ T lymphocytes was significantly elevated compared to CD3^+^CD4^+^ TIGIT^−^ T lymphocytes in SLE patients (P = 0.023). **d** The frequency of PD1-expressing CD3^+^CD4^+^ T lymphocytes was significantly increased in SLE patients compared to HCs (P = 0.013). **e** The frequency of PD1-expressing CD3^+^CD8^+^ T lymphocytes was significantly increased in SLE patients compared to HCs (P = 0.0024). **f** The frequency of PD1-expressing CD3^+^CD4^+^ TIGIT^+^ T lymphocytes was similar to CD3^+^CD4^+^ TIGIT^−^ T lymphocytes (P = 0.94). **g** The frequency of PD-L1-expressing CD3^+^CD4^+^ T lymphocytes was significantly increased in SLE patients compared to HCs (P = 0.0004). **h** The frequency of PD-L1-expressing CD3^+^CD8^+^ T lymphocytes was significantly increased in SLE patients compared to HCs (P < 0.0001). **i** The frequency of PD-L1-expressing CD3^+^CD4^+^ TIGIT^+^ T lymphocytes was similar to CD3^+^CD4^+^ TIGIT^−^ T lymphocytes (P = 0.27)

We next tried to determine whether the TIGIT expression level is related to activation markers of T lymphocytes. Data showed that the frequency of CD69-expressing CD3^+^CD4^+^ T lymphocytes was significantly elevated in patients with SLE compared to HCs (P = 0.019) (Fig. 2a). No significant difference was observed in the frequency of CD69-expressing CD3^+^CD8^+^ T lymphocytes between SLE individuals and HCs (Fig. 2). Furthermore, results showed the frequency of CD69-expressing CD3^+^CD4^+^ TIGIT^+^ T lymphocytes was significantly elevated compared to CD3^+^CD4^+^ TIGIT^−^ T lymphocytes in SLE patients (P = 0.023) (Fig. 2c). Our results suggest that the TIGIT expression level is related to the activation of CD4 T lymphocytes.

Evidences from our studies and other reports indicated that PD1/PD-L1 play an important role in SLE [17–19]. In the current study, our results showed that the frequencies of PD1-expressing CD3^+^CD4^+^ T lymphocytes and CD3^+^CD8^+^ T lymphocytes were significantly elevated in SLE patients compared to HCs (P < 0.05) (Fig. 2d, e). The frequencies of PD-L1-expressing CD3^+^CD4^+^ T lymphocytes and CD3^+^CD8^+^ T lymphocytes were also significantly elevated in SLE patients compared to HCs (P < 0.05) (Fig. 2g, h). We further investigated the relationship between the PD1/PD-L1 expression levels and TIGIT expression level in CD3^+^CD4^+^ T lymphocytes. Data showed the percentages of PD1^+^ and PD-L1^+^ cells were not significantly different between TIGIT^−^ and TIGIT^+^ CD3^+^CD4^+^ T lymphocytes (Fig. 2f, i).

### The frequency of TIGIT-expressing CD3^+^CD4^+^ T lymphocytes correlated with markers of autoimmune response and inflammation marker

**Fig. 3:**
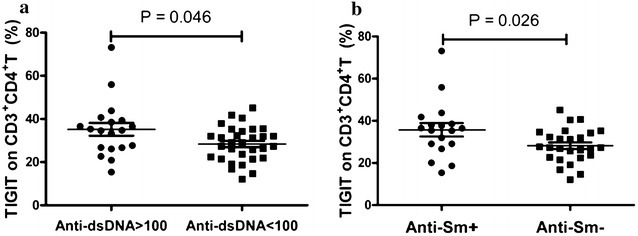
Correlation of frequency of TIGIT-expressing CD3^+^CD4^+^ T lymphocytes with autoantibody. **a** The frequency of TIGIT-expressing CD3^+^CD4^+^ T lymphocytes was significantly increased in SLE patients positive to anti-dsDNA (P = 0.046). **b** The frequency of TIGIT-expressing CD3^+^CD4^+^ T lymphocytes was significantly increased in SLE patients positive to anti-Sm (P = 0.027)

Anti-dsDNA and anti-ENAs, the hallmark antibodies of SLE, were determined and analyzed for their correlations with the frequency of TIGIT-expressing CD3^+^CD4^+^ T lymphocytes in this study. Data showed that 19 subjects were positive for anti-dsDNA in all recruited SLE patients. 44 patients were tested for anti-ENAs in all recruited SLE patients and 43 patients were positive for at least one anti-ENA. Although there is no obvious correlation between the frequency of TIGIT-expressing CD3^+^CD4^+^ T lymphocytes and anti-dsDNA level (data not shown), the frequency of TIGIT-expressing CD3^+^CD4^+^ T lymphocytes was significantly increased in patients with positive anti-dsDNA compared to patients with negative anti-dsDNA (P = 0.046) (Fig. 3a). Moreover, the correlation between the frequency of TIGIT-expressing CD3^+^CD4^+^ T lymphocytes and anti-ENAs including anti-SSA, anti-SSB, anti-Ro52, anti-Sm, anti-nRNP/Sm, anti-rRNP, and anti-nucleosome were also investigated in SLE patients. As shown in Fig. 3b, the frequency of TIGIT-expressing CD3^+^CD4^+^ T lymphocytes was significantly increased in patients with positive anti-Sm compared to patients with negative anti-Sm (P = 0.026). No obvious correlation was observed between the frequency of TIGIT-expressing CD3^+^CD4^+^ T lymphocytes and other anti-ENAs (data not shown).

Patients with SLE frequently have abnormal levels of inflammatory markers, such as IgG, C3, C4 and ESR. In order to investigate the correlations between the frequency of TIGIT-expressing CD3^+^CD4^+^ T lymphocytes and inflammatory markers, inflammation markers including serum IgG, C3, C4 and ESR were determined and analyzed for their correlations with the frequency of TIGIT-expressing CD3^+^CD4^+^ T lymphocytes in patients with SLE. In general, there was no correlation between increased frequency of TIGIT-expressing CD3^+^CD4^+^ T lymphocytes with serum IgG, C3, C4 and ESR (data not shown).

### The frequency of TIGIT-expressing CD3^+^CD4^+^ T lymphocytes correlated with urine protein

**Fig. 4:**
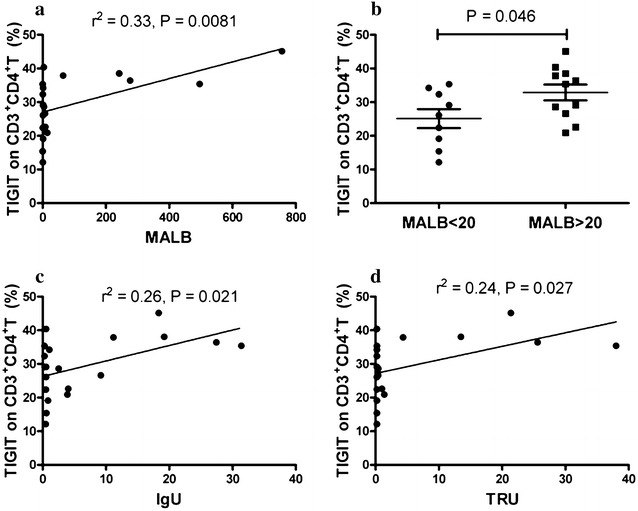
Correlation of frequency of TIGIT-expressing CD3^+^CD4^+^ T lymphocytes with urine protein. **a** The frequency of TIGIT-expressing CD3^+^CD4^+^ T lymphocytes in SLE patients correlated significantly with MALB (r^2^ = 0.33; P = 0.0081). **b** The frequency of TIGIT-expressing CD3^+^CD4^+^ T lymphocytes was significantly increased in SLE patients with MALB >20 mg/L (P = 0.046). **c** The frequency of TIGIT-expressing CD3^+^CD4^+^ T lymphocytes in SLE patients correlated significantly with IgU (r^2^ = 0.26; P = 0.021). **d** The frequency of TIGIT-expressing CD3^+^CD4^+^ T lymphocytes in SLE patients correlated significantly with TRU (r^2^ = 0.24; P = 0.027)

The results demonstrated that the frequency of TIGIT-expressing CD3^+^CD4^+^ T lymphocytes was correlated with markers of autoimmune response, such as anti-dsDNA. Anti-dsDNA is pathogenic autoantibody with the potential to result in renal damage [3, 20–22]. Thus, we investigated the correlation between the frequency of TIGIT-expressing CD3^+^CD4^+^ T lymphocytes and renal damage (characterized by proteinuria, hematuria, or >5 leukocytes/hpf excluding infection [23–25]). Disappointing, no obvious correlation was observed between the frequency of TIGIT-expressing CD3^+^CD4^+^ T lymphocytes and proteinuria, hematuria, pyuria in this study. Next, the correlations between the frequency of TIGIT-expressing CD3^+^CD4^+^ T lymphocytes and the quantificational levels of urine protein, including urine a_1_-M, urine microalbumin (MALB), urine IgG (IGU) and urine transferrin (TRU), were analyzed in 20 patients with SLE. As shown in Fig. 4, positive correlations between the frequency of CD3^+^CD4^+^ T lymphocytes and the levels of MALB (r^2^ = 0.33; P = 0.0081) (Fig. 4a), IgU (r^2^ = 0.26; P = 0.021) (Fig. 4c) and TRU (r^2^ = 0.24; P = 0.027) (Fig. 4d) were found. No obvious correlation was observed between the frequency of TIGIT-expressing CD3^+^CD4^+^ T lymphocytes and a_1_-M (data not shown). Moreover, we found that the frequency of TIGIT-expressing CD3^+^CD4^+^ T lymphocytes was significantly increased in patients with urine MALB levels >20 mg/L (P = 0.046) (Fig. 4b). And, the frequency of TIGIT-expressing CD3^+^CD4^+^ T lymphocytes trends to elevate in patients with higher a_1_-M, IgU and TRU (data not shown) respectively, but significant difference was not reached.

### The frequency of TIGIT-expressing CD3^+^CD4^+^ T lymphocytes correlated with disease activity of SLE

**Fig. 5:**
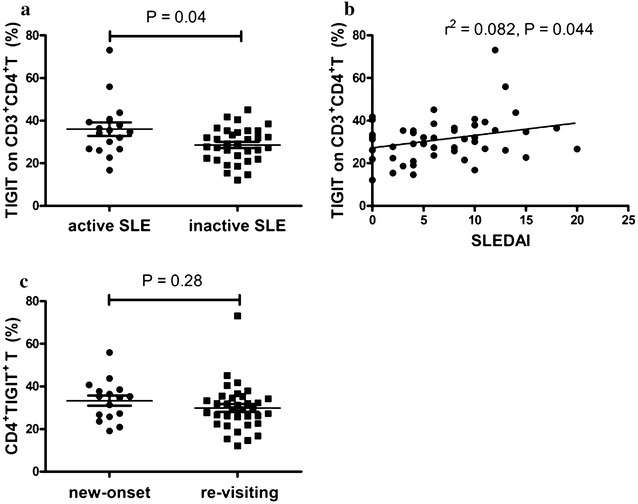
Correlation of frequency of TIGIT-expressing CD3^+^CD4^+^ T lymphocytes with disease activity. **a** The frequency of TIGIT-expressing CD3^+^CD4^+^ T lymphocytes in SLE patients was significantly increased in active SLE patients compared to inactive SLE patients (P = 0.04). **b** The frequency of TIGIT-expressing CD3^+^CD4^+^ T lymphocytes in SLE patients correlated significantly with SLEADI (r^2^ = 0.082; P = 0.044). **c** The frequency of TIGIT-expressing CD3^+^CD4^+^ T lymphocytes tends to be elevated in new-onset patients of SLE, but a significant difference was not reached (P = 0.28)

Our results demonstrated that the frequency of TIGIT-expressing CD3^+^CD4^+^ T lymphocytes was correlated with markers of autoimmune response. Some of these markers such as anti-dsDNA, is traditionally valuable for monitoring disease activity in patients with SLE [22]. Thus, patients with SLE were further classified as active and inactive patients according to the SLEDAI and the correlation between the frequency of TIGIT-expressing CD3^+^CD4^+^ T lymphocytes and disease activity was analyzed. Data showed that the frequency of TIGIT-expressing CD3^+^CD4^+^ T lymphocytes in patients with active SLE was significantly higher compared with patients with inactive SLE (P = 0.04) (Fig. 5a). Furthermore, there was a positive correlation between the frequency of TIGIT-expressing CD3^+^CD4^+^ T lymphocytes and the SLEDAI score (r^2^ = 0.082; P = 0.044) (Fig. 5b). These results thus demonstrated that the frequency of TIGIT-expressing CD3^+^CD4^+^ T lymphocytes was correlated with disease activity of SLE.

Subsequently, we compared the frequency of TIGIT-expressing CD3^+^CD4^+^ T lymphocytes between new-onset and re-visiting SLE patients. Data showed that the frequency of TIGIT-expressing CD3^+^CD4^+^ T lymphocytes tends to be elevated in new-onset patients, but a significant difference was not reached (Fig. 5c).

Although the frequency of TIGIT-expressing CD3^+^CD4^+^ T lymphocytes was associated with disease activity of SLE, it was not associated with clinical features of patients with SLE including fever, cutaneous manifestations, oral ulcer, alopecia, arthritis, Raynaud’s phenomenon, effusion, and hematologic disorder (data not shown).

## Discussion

Systemic lupus erythematosus (SLE) is a systemic autoimmune syndrome with unclear etiology. Costimulatory molecules were reported to play important roles in SLE [13, 26]. Program death-1 and program death-1 ligands (PD1/PD-L1), which have been proven to play a major role in suppressing immune response, were found to be elevated expressed on T cells of patients with SLE [17, 18]. T cell immunoglobulin domain- and mucin domain containing molecule-3 (Tim-3), a co-inhibitory type 1 transmembrane protein, has been identified to be expressed at high levels on T cells and associated with disease activity in SLE patients [27]. Cytotoxic T-lymphocyte-associated antigen-4 (CTLA-4), a member of the immunoglobulin superfamily that can downregulate T-cell function, was found to be increased on T cells, and CTLA-4 dysfunction has been identified as a potential cause for abnormal T-cell activation in patients with SLE [28]. In addition, CD40 ligand (CD40L) and many other inhibitory receptors could also be expressed on T cells in SLE [29, 30]. TIGIT is a newly identified inhibitory type 1 transmembrane protein expressed on immune cells. In this study, for the first time, we investigated the expression of TIGIT on neutrophils, monocytes, B and T lymphocytes from patients with SLE, and showed that the frequency of TIGIT-expressing CD3^+^CD4^+^ T lymphocytes was significantly increased in patients with SLE compared with HCs. Moreover, our research revealed that the frequency of TIGIT-expressing CD3^+^CD4^+^ T lymphocytes was associated with disease activity of SLE.

Lymphocytes were reported to play important roles in the development and progression of disease [31, 32]. Consistent with previous studies [33, 34], our study found a lower proportion of CD3^+^CD4^+^ T cells and higher proportion of CD3^+^CD8^+^ T cells in SLE patients. And, we found that the frequency of TIGIT-expressing CD3^+^CD8^+^ T lymphocytes was significantly elevated compared to CD3^+^CD4^+^ T lymphocytes. No significant difference was observed in the frequency of TIGIT-expressing CD3^+^CD8^+^ T lymphocytes between SLE patients and HCs, while the frequency of TIGIT-expressing CD3^+^CD4^+^ T lymphocytes were significantly elevated in patients with SLE compared to HCs. It supports the observations that SLE involves an imbalance of T cell subsets and that the abnormal expression of key signaling molecules on T lymphocytes plays an important role in SLE pathogenesis [35].

In addition to imbalance of T cell subsets, another characteristic of SLE is dysregulated activation of T lymphocytes. CD69 is an early cell activation marker that could show the quantity of active immune cells in the disease activity [36]. And, CD69 was usually used to evaluate the activation of T lymphocytes in SLE. Other reports [36, 37] and our results showed that the frequency of CD69-expressing CD3^+^CD4^+^ T lymphocytes was significantly elevated in patients with SLE. Moreover, we found that the frequency of CD69-expressing CD3^+^CD4^+^ TIGIT^+^ T lymphocytes was significantly elevated compared to CD3^+^CD4^+^ TIGIT^−^ T lymphocytes in SLE patients. Our results suggest that the TIGIT expression level is related to activation of CD3^+^CD4^+^ T lymphocytes. It supports the fact that the frequency of TIGIT-expressing CD3^+^CD4^+^ T lymphocytes was associated with disease activity of SLE and dysregulated activation of T lymphocytes involves in SLE pathogenesis.

Indeed, TIGIT was shown to be involved in the B cell regulation and antibodies production by regulating the interactions between T cells and follicular dendritic cells [38, 39]. It is well-known that SLE is a systemic autoimmune diseases characterized by elevated autoimmune antibodies, such as anti-dsDNA and anti-Sm. In this study, the serous levels of anti-dsDNA and anti-ENAs including anti-SSA, anti-SSB, anti-Ro52, anti-Sm, anti-nRNP/Sm, anti-rRNP, and anti-nucleosome, were determined and analyzed for their relationship with the frequency of TIGIT-expressing CD3^+^CD4^+^ T lymphocytes. Data showed that the frequency of TIGIT-expressing CD3^+^CD4^+^ T lymphocytes was significantly increased in patients with positive anti-dsDNA and anti-Sm, suggesting that TIGIT-expressing CD3^+^CD4^+^ T lymphocytes might be associated with autoimmune responses in SLE.

Anti-dsDNA is one of pathogenic autoantibodies which was reported to involve in renal damage [22]. So next we investigated the association between TIGIT-expressing CD3^+^CD4^+^ T lymphocytes and renal damage. Due to low compliance of renal biopsy, proteinuria, hematuria, Pyuria et al. were chosen to evaluate the renal damage. Results showed that the frequency of TIGIT-expressing CD3^+^CD4^+^ T lymphocytes was positively correlated with MALB. This supported the correlation between the frequency of TIGIT-expressing CD3^+^CD4^+^ T lymphocytes and renal damage of SLE patients. Subsequent results resulted from the SLEDAI classification of SLE patients confirmed that there was a positive correlation between the frequency of TIGIT-expressing CD3^+^CD4^+^ T lymphocytes and the SLEDAI score. But, TIGIT-expressing CD3^+^CD4^+^ T lymphocytes was not associated with other clinical features of patients with SLE including fever, cutaneous manifestations, oral ulcer, alopecia, arthritis, Raynaud’s phenomenon, effusion and hematologic disorder. Thus, we established the correlation between the frequency of TIGIT-expressing CD3^+^CD4^+^ T lymphocytes and disease activity in SLE.

TIGIT is an inhibitory costimulatory molecule that mediates inhibitory signal in immune cells [8]. Consistent with its inhibitory characteristics, the expression of TIGIT on NK cells was reported to be decreased in SLE patients [13]. Thus, from this point, the increased frequency of TIGIT-expressing CD3^+^CD4^+^ T lymphocytes in SLE seemed controversial to its function. However, evidences have also suggested that TIGIT might be involved in regulating B cell responses and promoting antibodies production [38, 39]. In this study, we found that SLE specific autoantibody levels, such as anti-dsDNA and anti-Sm, were positively correlated with the frequency of TIGIT-expressing CD3^+^CD4^+^ T lymphocytes. Thus, although the detail roles of TIGIT in SLE require further investigation, it seemed that besides functions as an inhibitory costimulatory molecule, TIGIT might plays other roles in SLE. Evidences [12, 38] from recent reports indicated that TIGIT could expressed on different CD3^+^CD4^+^ T lymphocytes subsets, such as T regulatory (Treg) cells and follicular helper T (Tfh) cells, and the functions of TIGIT on different T lymphocytes subsets were different. While the exact role of Tregs and Tfh cells in human SLE has yet to be established, recent data suggests that Treg cells and Tfh cells may be dysregulated in more active SLE patients [26, 40]. Future work is necessitated to clarify the roles and mechanisms of TIGIT expressing CD3^+^CD4^+^ T lymphocytes in SLE, especially, the roles and mechanisms of TIGIT expressing Treg cells and Tfh cells.

## Conclusions

To our knowledge, this is the first report on the characteristics of TIGIT-expressing CD3^+^CD4^+^ T lymphocytes in SLE. Additionally, our research established a correlation between the frequency of TIGIT-expressing CD3^+^CD4^+^ T lymphocytes and disease activity of SLE, which might improves our understanding of the roles of CD3^+^CD4^+^ T lymphocytes in SLE.

## Abbreviations

a_1_-M: urine a_1_-microglobulin
ANA: anti-nuclear antibodies
Anti-dsDNA: anti double-stranded DNA
Anti-SSA: anti-SSA antigen
Anti-SSB: anti-SSB antigen
APC: antigen-presenting cells
C3: complement 3
C4: complement 4
CD40L: CD40 ligand
CTLA-4: cytotoxic T-lymphocyte-associated antigen-4
ENAs: extractable nuclear antigens
ESR: erythrocyte sedimentation rate
HCs: healthy controls
ICOS: inducible T cell co-stimulator
IgG: immunoglobulin G
IGU: urine IgG
MALB: urine microalbumin
PD1: programmed death 1
PD-L1: programmed death ligand 1
PVR: poliovirus receptor
RNP: ribonucleoprotein
rRNP: ribosomal RNP
SLE: systemic lupus erythematosus
SLEDAI: SLE disease activity index
Sm: Smith
TIGIT: T cell immunoreceptor with Ig and immunoreceptor tyrosine-based inhibitory domains
Tim-3: T-cell immunoglobulin and mucin domain-containing protein 3
TRU: urine transferring
Tfh: follicular helper T cells
Treg: T regulatory cells
